# Editorial: The Role of Pentraxins: From Inflammation, Tissue Repair and Immunity to Biomarkers

**DOI:** 10.3389/fimmu.2019.02817

**Published:** 2019-12-03

**Authors:** Barbara Bottazzi, Cecilia Garlanda, Mauro Martins Teixeira

**Affiliations:** ^1^Humanitas Research Hospital, Rozzano, Italy; ^2^Department of Biomedical Sciences, Humanitas University, Rozzano, Italy; ^3^Department of Biochemistry and Immunology, Institute of Biological Sciences, Federal University of Minas Gerais, Belo Horizonte, Brazil

**Keywords:** pentraxins, inflammation, CRP—C-reactive protein, PTX3, SAP, acute phase protein

Pentraxins are a superfamily of highly conserved molecules characterized by a common structural motif, the so-called “pentraxin domain” (1, 2). C reactive protein (CRP), originally identified for its ability to bind the C-polysaccharide of *Streptococcus pneumoniae*, and serum amyloid P component (SAP) are the prototypes of the family and constitute the short pentraxin arm. The latter are 25 kDa secreted proteins characterized by a structural organization in which five (CRP) or ten (SAP) identical protomers are assembled in a pentameric symmetry. The long pentraxin PTX3, first identified in the early 90's as prototype of the long pentraxin family, is characterized by the presence of a long N-terminal domain unrelated to other proteins. Short and long pentraxins diverged from a common ancestor of all pentraxins, an events that occurs very early in evolution, given that members of the long pentraxin superfamily where identified in the most ancient vertrebate *Takifugu rubripes* (1).

CRP, SAP and PTX3 are multifunctional molecules mainly produced by inflammatory mediators and tissue injury. CRP is the most important acute phase protein in humans and is routinely measured to monitor human diseases. SAP contributes to amyloid formation and is possibly a therapeutic target. PTX3 is an essential mediator of innate resistance to selected pathogens of fungal, bacterial and viral origin, and is involved in regulation of inflammation, tissue remodeling and cancer.

This Research Topic, carried out with the support of the International Union of Immunological Societies (IUIS), wants to offer an overview of the main biological characteristics of these proteins, pointing to their essential role as regulators of the innate immune response and the possible translational implications.

The review from Pepys can be considered the grand opening of the Research Topic (Pepys). Pepys trace the history of short pentraxins from the discovery to their structural characterization, from the biological properties to the translational potential. In particular, it is shown how both CRP and SAP have become extremely useful as biomarker of human disease and as possible therapeutic targets in different pathological conditions, including amyloidosis and Alzheimer's disease (SAP), or myocardial and cerebral infarction (CRP).

The essential role that the members of the pentraxin superfamily exert in the innate immune response fully accounts for the strong evolutionarily pressure observed. Pathak and Agrawal describe the organisms where CRP has been found and the evolution of CRP from a constitutively expressed protein in arthropods to an acute phase molecule in humans. They also report the structural and biological similarities and differences among CRPs from different animals, while Ngwa and Agrawal describe the relationship between structure and function, in particular in relation to the anti-bacterial effect of CRP. Different structures are reported for CRP, with a native or a non-native pentameric protein and a monomeric molecule. Singh and Agrawal investigate the contribution of the different structural arrangements of CRP in relation to the atheroprotective role of the protein.

A main property of pentraxins is represented by the regulation of Complement activation. Two papers in this Research Topic are dealing with this important aspect of the regulation of inflammation (Ma and Garred, Haapasalo and Meri). The classical, the alternative and the lectin pathway of complement activation are all affected by interaction of members of the pentraxin family with their main initiating molecules (C1q, Ficolin-2, Mannose Binding lectin, CL-12) or regulators (Factor H and C4-binding protein). Ma and Garred underline the role of pentraxins in complement activation via crosstalk with both initiators and regulators of the classical and lectin pathways, while Haapasalo and Meri focus their review on the regulation of the alternative pathway. In both reviews, it is evidenced how the regulation of complement activity is an essential component of the role of CRP, SAP and PTX3 in immunosurveillance, anti-microbial immune response and immunologic homeostasis. In line with the general cross-talk between pentraxins and complement molecules, PTX3 has been shown to interact with C1q through the globular recognition domains (gC1q) modulating complement activity via the classical complement pathway. Bally et al. dissect the molecular determinants of this interaction, showing a key contribution of the B chain Arg residues that line the side of the gC1q heterotrimer, supporting the hypothesis that binding of C1q to targets through this region triggers efficient activation of the C1 complex.

Pentraxins can also directly bind to selected pathogens to act as opsonins and promote the removal of recognized microorganisms through phagocytosis. Lu et al. coworkers review the interaction of CRP, SAP and PTX3 with Fc receptors and describe the structural and functional characteristics of this interaction. The interaction of pentraxins with Fc receptors results in activation of cellular immune functions, similarly to Fc receptor activation by immune-complexes.

A unique characteristic of SAP is its ability to be deposited on amyloid fibrils, contributing to amyloid formation. Based on this observation, Pilling and Gomer describe in their review how SAP has been developed as possible therapeutic. SAP administration can inhibit fibrosis, an effect observed in preclinical studies as well as in small clinical trials with myelofibrosis patients. On the other hand, SAP depletion has a therapeutic potential for amyloidosis and can result in unleashing the innate immune system (Pilling and Gomer).

Doni et al. give a general overview on the structure and function of PTX3 and focus on the involvement of the molecule in sterile conditions of tissue damage and cancer, providing evidence that microbial and matrix recognition are evolutionarily conserved properties shared by humoral innate immunity molecules. They report that in models of tissue damage, PTX3 promotes tissue remodeling repair by interacting with fibrinogen/fibrin, as well as plasminogen (Plg), and favoring pericellular fibrinolysis. They also discuss the complexity of the roles of PTX3 in cancer, suggesting that PTX3 may have different functions on carcinogenesis depending on the tissue and cancer type. PTX3 is involved in tuning carcinogenesis through the modulation of cancer-related inflammation or angiogenesis or has a pro-tumorigenic function, by promoting tumor cell migration and invasion and macrophage infiltration (Doni et al.). One of the mechanisms underlying the involvement of PTX3 in tissue remodeling and cancer stems for its interaction with FGF2 and other members of the FGF family via its N-terminal domain, leading to inhibition of FGF-mediated angiogenic responses, in particular in FGF-dependent tumors and FGF2-mediated smooth muscle cell proliferation and artery restenosis. Presta and Foglio discuss this property of PTX3 and present the first low molecular weight pan-FGF trap able to inhibit FGF-dependent tumor growth and neovascularization, identified based on the FGF2/PTX3 interaction, and the implications for its development in FGF-mediated clinical conditions. de Oliveira et al. discuss the role of PTX3 in ischemia and reperfusion injury (IRI), a condition associated with increased expression of this pentraxin in response to DAMPS and inflammatory cytokines. In condition of sterile IRI, such as acute myocardial infarction or kidney, lung and brain IRI, PTX3 deficiency results in worse outcome. Regulation of P-selectin-dependent neutrophil recruitment in damaged tissues and tuning of complement activation and inflammation by PTX3 are among the most relevant mechanisms proposed. On the contrary, PTX3 was shown to have a clear deleterious role in intestinal IRI, a condition associated with significantly more systemic inflammation and remote damage than in the other models of IRI, potential loss of the intestinal barrier and bacterial translocation (de Oliveira et al.).

The generation of PTX3-deficient mice provided the first evidence that this molecule plays a non-redundant role in female fertility. Camaioni et al. discuss the studies performed in this field demonstrating that PTX3 is synthesized before ovulation by cells surrounding the oocyte and actively participates in the organization of the hyaluronan-rich provisional matrix required for successful fertilization. These results are relevant in humans since PTX3 polymorphisms have been associated with female fertility, in terms of dizygotic twinning and number of children given birth during the lifetime (3). It has been proposed that PTX3 may act as a biomarker of oocyte quality, and its systemic levels, determined by genetic variations and/or low-grade chronic inflammation, may affect the growth and development of the follicle and affect the incidence of ovarian disorders (Camaioni et al.).

In line with the role of the short pentraxin CRP as a systemic biomarker and independent predictor of adverse cardiovascular events, such as acute myocardial infarction, stroke, and peripheral artery disease, the involvement of PTX3 in cardiovascular diseases (CVD) has been investigated in mice and humans. Ristagno et al. discuss data on animal CVD models indicating that PTX3 can have cardioprotective and atheroprotective roles by regulating inflammation. In addition, data collected in several clinical settings indicate that PTX3 is a potential biomarker of CVD. PTX3 plasma levels rise rapidly in acute myocardial infarction, heart failure and cardiac arrest, reflecting the extent of tissue damage and predicting the risk of mortality. Along the same line and based on the expression of PTX3 by endothelial cells (Ramirez et al.), discuss the association between PTX3 concentration and autoimmune vasculitis, showing that systemic lupus erythematosus (SLE), ANCA-associated systemic small vessel vasculitides, giant cell arteritis and Takayasu's arteritis were all associated with increased PTX3 plasma concentration, which correlated with disease activity, acute phase reactants and prednisone treatment. Their study suggests that high levels of PTX3 in the systemic circulation can be used to identify the risk of vascular involvement in systemic immune-mediated diseases. It has been shown that SLE patients display high frequencies and titers of anti-PTX3 antibodies, which are inversely correlated with Lupus nephritis (LN) occurrence, suggesting an immunomodulatory capacity of anti-PTX3 antibodies. Gatto et al. describe the identification and characterization of peripheral B cells recognized by PTX3 present in SLE patients and healthy donors, but absent in LN patients, and suggest a potential immune regulatory role or protective function of these B cells.

Finally, Trojnar et al. investigated the involvement of PTX3 and CRP in thrombotic microangiopathies, such as typical and atypical hemolytic uremic syndrome, secondary thrombotic microangiopathies and thrombotic thrombocytopenic purpura. They found that both PTX3 and CRP levels were elevated in the acute phase of thrombotic microangiopathies. In contrast with CRP, PTX3 levels were associated with patient survival, and signs of complement consumption.

Identification of sepsis biomarkers allowing early stratification and recognition of patients at higher risk of death is crucial. PTX3 has been proposed as a promising biomarker candidate in sepsis patients since PTX3 plasma concentration increase and persistence has been positively associated with severity and mortality. Albert Vega et al. elucidated that despite their immune dysfunctions, circulating cells were responsible for the maintenance of PTX3 concentration in the blood of severe sepsis patients.

PTX3 has been previously described to bind both human and murine cytomegalovirus (CMV) and mediate several host antiviral mechanisms. Campos et al. show the contribution of genetic variation in donor PTX3 to the risk of CMV reactivation in patients undergoing allogeneic hematopoietic stem-cell transplantation. This result suggests that donor PTX3 allelic variants can predict the risk of CMV reactivation in this clinical setting, similarly to what reported on invasive aspergillosis in hematopoietic stem-cell transplanted patients (4).

This Research Topic describes the pleiotropic functions of pentraxin family members and suggests the complexity of their involvement in modulating innate and inflammatory responses. The potential contradictory roles of these molecules in health and disease depends on the disease context, the cellular source, or the levels of protein released. Deciphering more clearly the multifaceted functional roles of pentraxins, and in particular PTX3, in physiology and disease may facilitate the development of targeted therapeutic approaches in various clinical conditions.

## Author Contributions

All authors listed have made a substantial, direct and intellectual contribution to the work, and approved it for publication.

### Conflict of Interest

The authors declare that the research was conducted in the absence of any commercial or financial relationships that could be construed as a potential conflict of interest.

## References

[1] Garlanda C Bottazzi B Bastone A Mantovani A. Pentraxins at the crossroads between innate immunity, inflammation, matrix deposition, and female fertility. Annu Rev Immunol. (2005) 23:337–66. 10.1146/annurev.immunol.23.021704.115756 15771574

[2] Casas JP Shah T Hingorani AD Danesh J Pepys MB. C-reactive protein and coronary heart disease: a critical review. J Intern Med. (2008) 264:295–314. 10.1111/j.1365-2796.2008.02015.x 18823504

[3] Sirugo G Edwards DR Ryckman KK Bisseye C White MJ Kebbeh B. PTX3 genetic variation and dizygotic twinning in the Gambia: could pleiotropy with innate immunity explain common dizygotic twinning in Africa? Ann Hum Genet. (2012) 76:454–63. 10.1111/j.1469-1809.2012.00723.x 22834944 PMC3731069

[4] Cunha C Aversa F Lacerda JF Busca A Kurzai O Grube M. Genetic PTX3 deficiency and aspergillosis in stem-cell transplantation. N Engl J Med. (2014) 370:421–32. 10.1056/NEJMoa1211161 24476432

